# Primary Peritonitis: An Index Case of *Mycoplasma hominis* Infection in a Healthy Female

**DOI:** 10.1155/2018/4587801

**Published:** 2018-02-06

**Authors:** Sabrina Drexel, Daniel Tseng

**Affiliations:** ^1^Oregon Health and Science University, 3181 SW Sam Jackson Park Rd., Portland, OR 97239, USA; ^2^Legacy Good Samaritan Hospital, 1015 NW 22nd Ave., Portland, OR 97210, USA

## Abstract

**Introduction:**

Primary peritonitis in healthy immunocompetent individuals is rare. Several case reports of *Streptococcus* species causing peritonitis have been described. Here, we present the first case of *Mycoplasma hominis* as the cause of primary peritonitis in a healthy woman.

**Case Report:**

A 42-year-old female with history of uterine fibroids was admitted with abdominal pain and intraperitoneal fluid of unknown etiology. She was initially managed nonoperatively and empirically treated with broad spectrum antibiotics. Blood and urine cultures were unrevealing. Increasing abdominal pain and peritoneal fluid prompted diagnostic laparoscopy which revealed a dense fibrinous exudate covering the entire peritoneal cavity. Peritoneal fluid and biopsies were sent for cytology and culture. The peritoneal fluid was eventually sent for 16 s ribosomal analysis, which discovered *Mycoplasma hominis* RNA. Her antibiotics were narrowed, and she eventually made a full recovery.

**Discussion:**

*M. hominis* is a rare source of systemic infection but has been known to colonize the urogenital tract and cause localized infections. This is the first presentation of *M. hominis* causing primary peritonitis in a healthy immunocompetent female. Multidisciplinary management of these patients is critical to achieve a timely diagnosis. Surgical exploration is often unavoidable to rule out secondary peritonitis.

## 1. Introduction

Peritonitis is a clinical syndrome that is derived from peritoneal irritation. This diagnosis may be suspected by abdominal exam findings such as voluntary and involuntary guarding, localized tenderness, and rebound tenderness. Peritonitis may occur as a primary process seen in patients with severe liver disease, immunocompromised states, nephrotic syndrome, or heart disease. However, surgeons often are involved in the diagnosis and management of peritonitis secondary to complications involving visceral organs. These patients typically require surgical intervention. In contrast, primary peritonitis is often diagnosed with paracentesis and treated with antibiotics. The most common organisms that cause primary peritonitis are gram-negative enteric bacteria (60%), including *Escherichia coli* and *Klebsiella pneumoniae*. Gram-positive cocci account for 25%, in which *Streptococcus* is most commonly isolated [1].

Primary peritonitis is an extremely rare entity in healthy adults. There are numerous case reports of Group A Streptococcus (GAS) and *Streptococcus pneumoniae* causing primary peritonitis in young previously healthy individuals [1–3]. Primary peritonitis with *Mycoplasma hominis* has been described in the renal transplant population [4–6]; however, there is no known reported case of primary peritonitis caused by *Mycoplasma hominis* in a healthy nonimmunocompromised adult. To our knowledge, we present the first case of *Mycoplasma hominis* peritonitis in an otherwise healthy young female.

## 2. Case Report

**Figure 1:**
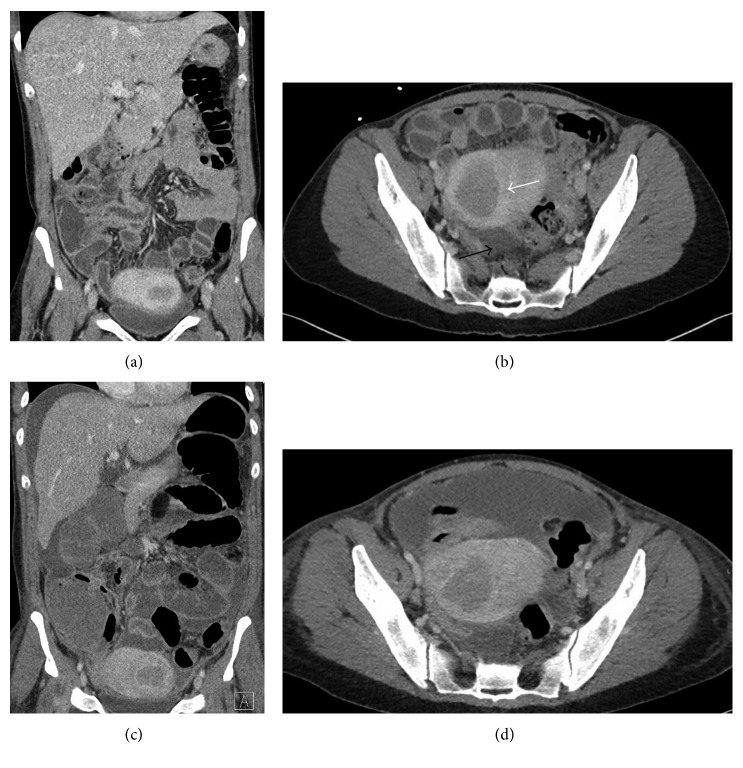
Initial CT scan upon presentation (a, b) with uterine fibroids (white arrow) and minimal free fluid (black arrow). CT scan four days after admission with worsening ascites (c, d).

A 42-year-old female was seen in the emergency department with three days of severe abdominal pain and sepsis. Medical history included menometrorrhagia due to uterine fibroids and previous endometrial ablation in 2015. She used a contraceptive vaginal ring for three months prior and Provera for 2 days prior to presentation for persistent vaginal bleeding. Physical examination revealed the following vital signs: temperature 39.2°C; blood pressure 136/78 mmHg, pulse 132 beats/min, and respiratory rate 16 breaths/min. Oxygen saturation was 100% on room air. Physical exam revealed tenderness throughout the lower abdomen with voluntary guarding. Labarotory data revealed WBC of 12,000/*µ*L, Hgb of 7.8 gm/dL and Hct of 25.7, sodium of 127 mmol/L, and chloride of 96 mmol/L. Lactate was normal at 1.5 mmol/L. Initial CT scan showed uterine masses, consistent with known fibroids, and minimal pelvic fluid (Figure 1).

She was initially admitted to the medical service for sepsis. Given her lower abdominal pain, Gynecology was consulted for presumed pelvic inflammatory disease (PID). She was started on empiric antibiotics for PID (ceftriaxone IV 1 g every 24 hours, azithromycin IV 500 mg every 24 hours, and metronidazole IV 500 mg every 8 hours). Blood, urine, and stool cultures were sent upon admission, all of which were negative.

**Figure 2:**
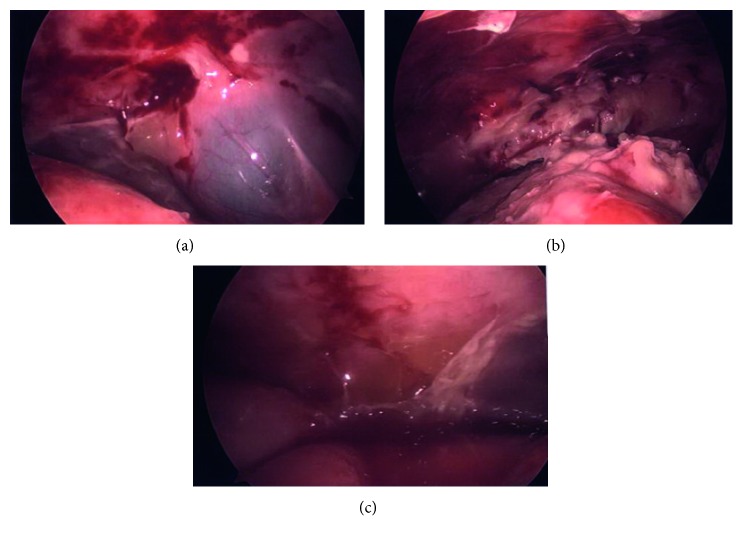
Diagnostic laparoscopy findings: inflamed peritoneum with densely adhered gallbladder to liver (a); fibrinous exudate throughout the peritoneal cavity (b); gelatinous ascites (c).

Her pain continued to progress with rising leukocytosis to 20,000/*µ*L. A repeat CT scan on hospital day four showed interval development of ascites (Figure 1). Surgical consultation was obtained, and she was found to have an acute abdomen. She was taken for a diagnostic laparoscopy, which revealed a dense fibrinous exudate and significant ascites in all four quadrants of the abdomen (Figure 2). Multiple peritoneal biopsies were taken, and peritoneal fluid was sent for gram stain, cultures, and cytology.

Postoperatively, carcinoembryonic antigen (CEA) and CA-125 levels were obtained. CEA was within the normal range, and CA-125 was mildly elevated at 56.8 U/mL (normal 0–35.0 U/mL). Her WBC fluctuated between 15,000 and 25,000/*µ*L. The peritoneal fluid gram stain did not reveal any bacteria. Cytology revealed rare mesothelial cells and abundant neutrophils but no malignant cells. Final pathology report stated the presence of benign fibromembranous tissue with severe acute inflammation and extensive necrosis, consistent with “severe necrotizing acute peritonitis.”

Given the extensive inflammatory reaction, infectious disease was consulted. Group A Strep (GAS) peritonitis was suspected, and recommendations were made to continue ceftriaxone at 1 g IV every 24 hours in addition to one dose of clindamycin 600 mg IV. Azithromycin was added to ceftriaxone three days later (IV 500 mg every 24 hours). A streptolysin O antibody (ASO) titer was sent but came back normal at 61 I U/mL (normal range 0–330 IU/mL), arguing against GAS peritonitis. Ceftriaxone was stopped, and azithromycin was transitioned to oral 500 mg daily.

**Figure 3:**
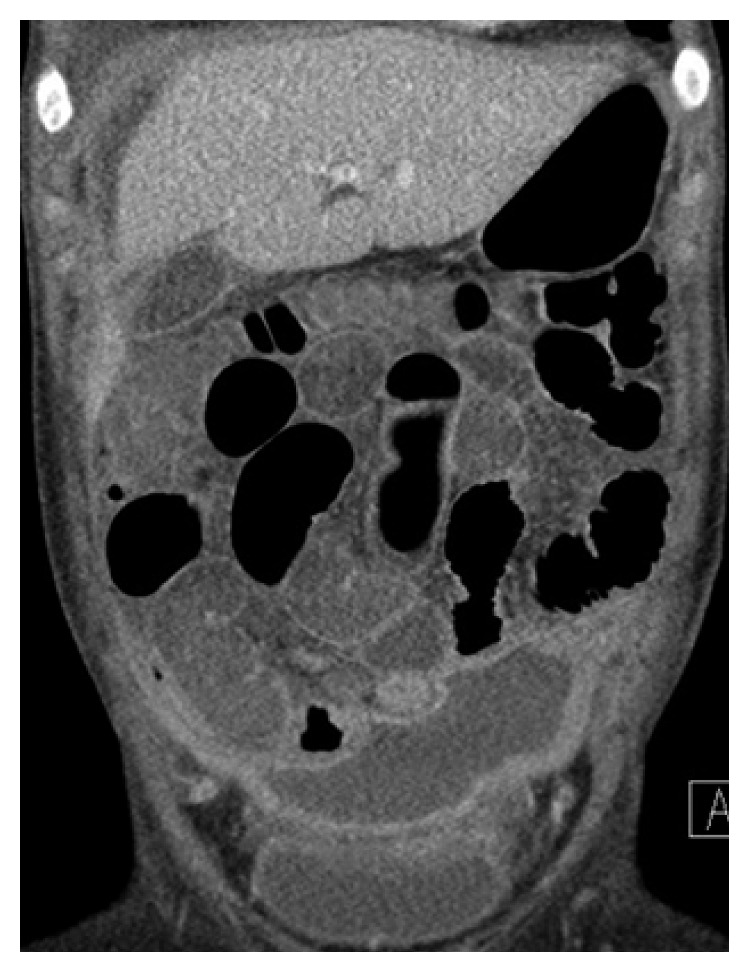
CT scan nine days after surgery with persistent ascites and peritoneal enhancement.

She continued to have persistent abdominal pain and anorexia. A repeat CT scan obtained nine days after surgery (Figure 3) showed peritoneal enhancement and several fluid collections. A diagnostic paracentesis was performed, and the peritoneal fluid was sent to an outside institution for 16S ribosome analysis, to isolate bacterial RNA.

Her abdominal pain and leukocytosis slowly improved. She continued to have severe anorexia and food aversion, despite reassuring physical exam findings. On hospital day 23, she was discharged home on oral azithromycin. Several days after discharge, 16S ribosome testing revealed *Mycoplasma hominis* RNA within the peritoneal fluid. Her outpatient antibiotic was changed to oral doxycycline 100 mg twice daily for three months. She was closely followed as an outpatient and gradually demonstrated clinical improvement. She remains well ten months after hospitalization.

## 3. Discussion

This is the first known case of *Mycoplasma hominis* primary peritonitis in an otherwise healthy female. In our case, detection of this organism proved to be extremely difficult. Standard analysis of blood, peritoneal fluid, and peritoneal biopsy cultures was unable to detect this organism. In contrast, other recognized causes of primary peritonitis in healthy women, such as *Streptococcus pneumoniae* and Group A Streptococcus, typically have positive cultures, making organism identification more straightforward [1–3].

*Streptococcus pneumoniae* has been described as a cause of primary peritonitis, especially in the pediatric population [3, 7, 8]. These patients typically present with an acute abdomen, and surgery is often pursued for presumed secondary peritonitis. A retrospective review of 27 case studies from 1966–1997 showed all patients had either positive blood or peritoneal cultures [9]. The patients were majority female, and the most common source of infection appeared to be the genital tract.

Group A Streptococcus (GAS) typically causes upper respiratory infections, although thirty-eight case reports for GAS primary peritonitis have been cited [2]. Most of these patients were female who presented with fevers and significant abdominal pain. All but one patient received exploratory surgery due to concern for secondary peritonitis, even despite negative imaging. 83% of these patients had positive blood cultures. In a more recent review of the 21st century case reports on GAS peritonitis, the authors noted none of these patients had free air on imaging, arguing against perforated viscus as the cause of peritonitis. Again, the majority of patients underwent exploratory surgery (92.1%). They conclude GAS peritonitis may not be as rare as previously suspected [3].

In general, *M. hominis* is unlikely to cause systemic infection, but it does colonize the urogenital tract and can generate localized infections. Up to 50% of sexually active women are colonized with *M. hominis* in the cervix or vagina [10]. The *Mycoplasma* species is extremely small and lacks a peptidoglycan cell wall; therefore, it does not appear on gram stain, making it particularly difficult to identify using traditional techniques. The most reliable test for detection of *M. hominis* is the 16S ribosomal RNA gene by PCR [11], although multiplex real time PCR tests are also being developed [12]. However, these tests are not available at all institutions and often takes days to weeks to obtain results. The lack of a peptidoglycan cell wall also makes *M. hominis* not susceptible to most broad spectrum antibiotics, including penicillins, cephalosporins, or carbapenems [13]. The treatment of choice is tetracyclines or fluoroquinolones [14].

*M. hominis* has been known to cause genitourinary inflammatory diseases in adults including pyelonephritis, pelvic inflammatory disease, bacterial vaginosis, and postpartum endometritis. It has a different spectrum of disease in neonates and infants [15]. In this sentinel case, the mode and route of infection is unclear. No vaginal or cervical cultures were taken during her hospitalization to investigate urogenital colonization of *M. hominis*, but this seems to be the most likely source. It remains unknown whether the patient's contraceptive vaginal ring, large uterine fibroids, and previous myometrial ablations played any role in her increased susceptibility.

In all cases of peritonitis, broad spectrum antibiotics should be started as soon as possible [16]. Surgery often remains the mainstay in diagnosis and treatment of patients with peritonitis. In this situation, primary bacterial peritonitis from a multitude of different organisms may need to be considered. A multidisciplinary approach with consultation of an infectious disease specialist is recommended early in the course of a patient with unclear source of peritonitis. Diligence to ensure there is no other cause for peritonitis is critical to obtain source control, and exploratory surgery is often unavoidable.
